# Epidemiological Features of Hand, Foot and Mouth Disease Outbreaks among Chinese Preschool Children: A Meta-analysis

**Published:** 2018-09

**Authors:** Xiao-Fang WANG, Jiao LU, Xiao-Xia LIU, Ting DAI

**Affiliations:** 1. Division of Preschool Health, Shanghai Normal University, Shanghai, China; 2. Dept. of Pediatrics, Shanghai General Hospital, Shanghai Jiao Tong University, Shanghai, China; 3. Dept. of Library, Shanghai Normal University, Shanghai, China; 4. Dept. of Public Health, Xuhui Central Hospital, Shanghai, China

**Keywords:** Hand, Foot and mouth disease, Preschool, Outbreak, Meta-analysis, Prevention

## Abstract

**Background:**

Hand-foot-mouth disease (HFMD) is a widespread communicable disease and has caused large epidemics in many countries. This meta-analysis aimed to analyze and evaluate the epidemiological features of HFMD outbreaks in Chinese preschools.

**Methods:**

Literature review was based on PubMed, Chinese National Knowledge Infrastructure (CNKI) and Wanfang databases from 2008 to 2015. The temporal, spatial and demographic parameters were summarized and analyzed.

**Results:**

Overall, 19 studies with a total of 11269 HFMD cases were selected for data synthesis and analysis. April, May, June and July were detected as the peak months of HFMD outbreaks, with the pooled rate of 21% (95% CI: 12%–34%), 23% (95% CI: 19%–27%), 20% (95% CI: 17%–24%) and 11% (95% CI: 7%–15%). Urban areas were at a higher risk of suffering from HFMD outbreaks than rural areas, with the pooled rate of 65% (95% CI: 48%–78%) and 35% (95% CI: 22%–52%) respectively. The constituent ratio of children aged 37–48 months is the highest, accounting for 46% (95% CI: 39%–53%) of the total cases during HFMD outbreaks. The pooled rate of male cases (60%) was higher than that of female cases (40%).

**Conclusion:**

Month, residence, age, and gender may be early risk factors for potential HFMD outbreaks. Before the advent of peak months from Apr to Jul each year, measures should be taken to prevent the HFMD outbreaks among preschool children in China. Preschools located in urban areas should take priority over special prevention. HFMD surveillance should preferentially focus on children aged 37–48 months, especially boys in preschools.

## Introduction

Hand, foot, and mouth disease (HFMD) is an acute communicable disease mostly happened to children. It is caused by viruses belonging to the Enterovirus genus, such as Coxsackievirus A16 (CA16) and Enterovirus71 (EV71) (1). Clinical symptoms usually include fever, malaise, rashes on hands and feet, and oral vesicles. Of note, severe and even life-threatening complications can develop rapidly in young children, such as acute pulmonary edema, cardiopulmonary failure, aseptic meningitis, encephalitis and acute flaccid paralysis (2). HFMD is transmitted from person to person by direct contact with respiratory droplets, open and weeping skin vesicles, blister fluid from mouth, feces, or through exposing to a contaminated environment (3).

In the past three decades, HFMD epidemic has been continuously observed across the world. Recently, HFMD has become an increasing burden in the Asia-Pacific Region including Japan, Malaysia, Singapore, Viet, China etc. (4–8). In 2008 the largest Asia-Pacific pandemic was reported in China, posing a big threat to public health. Then, HFMD was designated as a C-class notifiable communicable disease by the Ministry of Health of China on 2 May 2008. The incidence of HFMD increased rapidly from 86.59/100,000 in 2009 to 203.16/100,000 in 2014 (9). HFMD has been responsible for 350–900 reported deaths annually, predominantly among young children, over the period of 2010–2012 (10). Currently, the nationwide mortality of HFMD remains the highest in all C-class communicable diseases, of which the proportion is over 90% in peak seasons (11).

HFMD outbreaks among Chinese preschool children occurred continuously since 2008 (12). A HFMD outbreak was defined in accordance with the criteria of the 2009 prevention and control guideline for HFMD issued by the Ministry of Health of the People’s Republic of China: (1) 5 or more cases of HFMD in a school within 1 week; (2) 2 or more cases of HFMD in a classroom within 1 week (13). HFMD outbreaks among preschool children have become an important public health problem in China.

We performed a meta-analysis to evaluate the epidemiological features of HFMD outbreaks among preschool children. We assessed the spatio-temporal distribution of HFMD outbreaks, to identify early risk factors and provide reference for future surveillance and prevention. The results will be helpful to effectively contain the spread of HFMD.

## Methods

### Search strategy

We follow the preferred reporting items for meta-analysis of observational studies in epidemiology (MOOSE) (14). A systematic search strategy was developed by a research librarian and two study investigators, to ensure that all relevant studies under review were appropriate to use with preschool children in China. PubMed, Chinese National Knowledge Infrastructure (CNKI), and Wanfang databases were queried from 2008 to 2015. The search was conducted by using the following free-text terms and medical subject headings: “hand foot and mouth disease”, “HFMD”, “outbreak”, “pre-school”, “kindergarten”, “daycare center”, “nursery” and “China”. In addition, Google and Google Scholar searches were conducted as a means of identifying pertinent gray literature.

### Inclusion and exclusion criteria

Inclusion criteria for studies were as follows: (1) published in Chinese or English; (2) including information about epidemiology of HFMD outbreaks. (3) Studies including HFMD outbreaks that occurred in preschool settings rather than at other places. Studies were excluded if they met the following criteria: (1) not an original study, such as review papers; (2) case series which reported less than 3 outbreaks; (3) If the study was reported in duplicate, the article published with more cases were included.

### Study selection

Two reviewers reviewed the studies respectively, screening article titles and abstracts for possible inclusion. If the information is not sufficient enough to make a decision on whether the study should be included or not, the full text of the article was further reviewed. The results were checked and discussed by two reviewers to agree upon a final list of included studies.

### Data extraction and analysis

Data from eligible studies were extracted separately by two reviewers. For each included study, we extracted the following data from original publications: authors, year of publication, study location (province and city), study period, residence (urban or rural area), number of HFMD outbreaks in each month, age of the patients, total number of HFMD cases and number of HFMD outbreaks in each study. Disagreements between the two reviewers during data extraction were reconciled by a third investigator.

A published systematic meta-analysis technique was used to calculate the pooled parameters of HFMD outbreaks from all eligible studies. Meta-analysis was carried out by using the software in R version 3.2.1, downloaded from the Comprehensive R Archive Network (CRAN, https://cran.r-project.org/). Proportion was automatically selected as a summary statistic in R software to enable forest plots. Pooled proportion and 95% confidence intervals were obtained by random or fixed effects model determined by *I*^2^. Pooled rate was gotten from pooled proportion multiplied by 100%.

We assessed the temporal, spatial and demographic distribution trends of HFMD outbreaks, with respect to month, region, age and gender, etc. For monthly distribution analysis, proportion represents the ratio of the number of HFMD outbreaks in targeted months to the total number of outbreaks in a year. For regional distribution analysis, proportion represents the ratio of the number of HFMD outbreaks in urban or rural areas to the total number of outbreaks. For age distribution analysis, proportion represents the ratio of the number of HFMD cases in each age group to the total number of HFMD cases. In addition to age distribution, impact of gender was also analyzed as part of the demographic distribution assessment. Proportion represents the ratio of the number of boy or girl cases to the total number of HFMD cases.

The statistical heterogeneity across the studies was estimated by the Chi-square based Q test (*P*<0.10 indicating statistical significance. The *I*^2^ statistic for each analysis was calculated. Subgroup analyses were conducted to investigate the source of high heterogeneities (15). Finally, the random effect model would be used if the heterogeneities could not be reduced by subgroup analyses. Egger’s test was used to assess the publication bias, and a *P*-value of <0.05 was considered as statistically significant (16).

## Results

### Literature search and studies characteristics

**Fig. 1:**
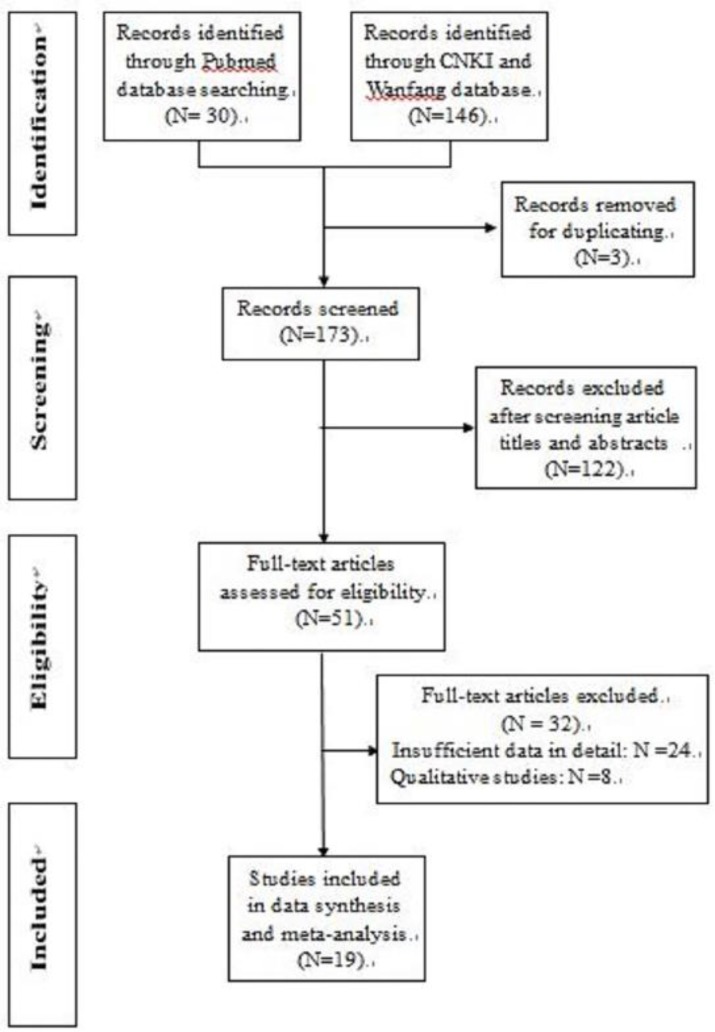
Flowchart of study selection

The results of the study selection process are shown in Fig. 1. The initial electronic search strategy identified 176 studies for review. In total, 51 potentially relevant articles were selected for full-text assessment. After a detailed evaluation, 19 original studies (17–35) remained for the final meta-analysis. A manual search of the reference lists of these studies did not yield any new eligible studies. Table 1 provides a descriptive summary of the included studies.

**Table 1:** Characteristics of included studies

***First author***	***Published year***	***Study period***	***Study location***	***Number of HFMD cases***	***Number of HFMD outbreak***
Li M (17)	2012	2011	Licheng	266	45
Kong ZF (18)	2014	2012	Ninhai	861	153
Wang RQ (19)	2013	2009	Changping	103	7
Zhang ZW (20)	2014	2012	Dongguan	130	7
Li Z (21)	2013	2012	Jinan	734	214
Tian L (22)	2011	2009	Shanghai	2993	672
Wang LJ (23)	2013	2010	Beijing	83	24
Ye ZW (24)	2014	2012	Zhuhai	1203	148
Xv JJ (25)	2013	2012	Yancheng	804	75
Chen HQ (26)	2010	2009	Jinan	87	5
Gu YG (27)	2010	2009	Jinin	127	28
Hu QP (28)	2015	2012	Pudong	462	79
Wang J (29)	2013	2010	Shanghai	219	40
Zhang CD (30)	2013	2010–2011	Lianyungang	225	52
Zhao ZR (31)	2013	2010–2011	Maanshan	1874	291
Xiong JG (32)	2012	2010	Dongguan	190	16
Li JY (33)	2012	2010	Foshan	345	65
Wang JP (34)	2013	2010	Yidu	137	27
Liu Y (35)	2015	2009–2013	Dandong	426	80

Overall, 2028 HFMD outbreaks including total 11269 HFMD cases were analyzed. There was no evidence for publication bias using the Egger’s test.

### Monthly distribution and peak months

The monthly distribution trends of HFMD outbreaks were investigated. The corresponding results are presented in Table 2. The pooled values of HFMD outbreaks from Jan to Dec were described in detail. HFMD outbreaks occurred year-round in China. During one year, the pooled rate of HFMD outbreaks was at the lowest level in Jan and Feb, with the numeral value of 2%. Then the rate began to increase in Mar and the numeral value was 7%. The pooled rate reached the highest level in the period of Apr to Jul, which was 21%, 23%, 20%, and 11% respectively. From Aug to Oct, the pooled rate remained the same (7%). In Nov the rate grew slightly to 8%. Finally, it dropped to 4% in Dec.

**Table 2:** Monthly distribution of HFMD outbreaks

***Month***	***Pooled rate (%)***	***95% CI (%)***	***Heterogeneity (%)***	***P***
January	2	1–5	0	0.78
February	2	1–4	0	0.89
March	7	4–12	33.20	0.14
April	21	12–34	79.10	<0.01
May	23	19–27	0	0.70
June	20	17–24	0	0.94
July	11	7–15	18.30	0.27
August	7	5–10	0	0.98
September	7	5–9	0	0.99
October	7	5–10	0	0.86
November	8	5–13	24.90	0.21
December	4	2–6	0	0.91

Peak months from Apr to Jul were observed. Over this consecutive period, the pooled rate of HFMD outbreaks was 72% (95% CI: 64%–79%), obviously higher than the sum of the rest months.

**Fig. 2:**
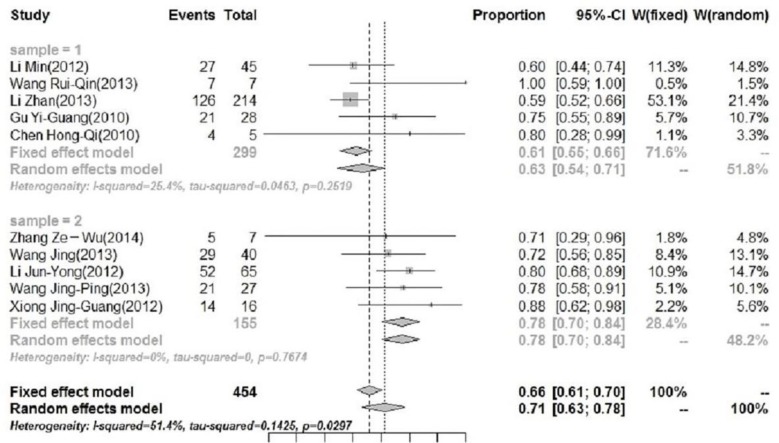
Monthly distribution of HFMD outbreaks in the period of April to July

However, the heterogeneity was significant (*I*^2^=54.2%; *P*=0.02). To eliminate the heterogeneity between studies, subgroup analyses were conducted for sample size, geographical regions, and study year. Finally, geographical difference was found to be the heterogeneous source. Southern China (sample 2) has higher pooled rate of HFMD outbreaks than Northern China (sample 1) during peak months, and the numeral value was 78% (95% CI: 70%–84%) and 61% (95% CI: 55%–66%) respectively (Fig. 2).

### Regional Distribution in Urban and Rural Areas

As for urban areas, the pooled rate of HFMD outbreaks is high, with the numeral value of 65% (95% CI: 48%–78%). While in rural areas the rate is lower, which was 35% (95% CI: 22%–52%). There was high heterogeneity between studies (*I*^2^=89.1%; *P*<0.0001) which could not be reduced by subgroup analyses. Therefore, the random effect model was used.

### Age distribution Trends

All the HFMD cases in each study were divided into four age groups, including 0–36 months, 37–48 months, 49–60 months and 61–72 months. Significant differences were observed between each age group. The pooled rate of HFMD cases was the highest in the age group 37–48 months and the numeral value was 46% (95% CI: 39%–53%). Next was the age group 49–60 months, with the pooled rate of 28% (95% CI: 25%–33%). As for the age group 61–72 months, the pooled rate was relatively low, which was 14% (95% CI: 10%–19%). The lowest was the age group 0–36 months, with the pooled rate of 9% (95% CI: 5%–15%).

### Impact of gender

**Fig. 3:**
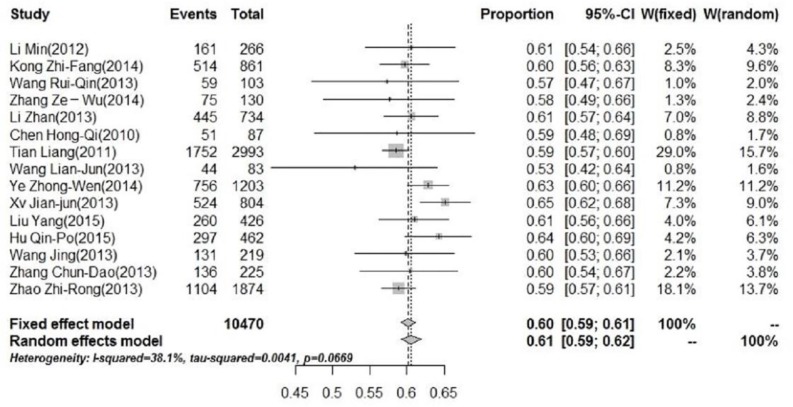
Forest plot of the constituent ratio for male cases

In addition to age distribution, impact of gender was also analyzed as part of the demographic distribution assessments. The pooled rate of male cases was 60% (95% CI: 59%; 61%). Fig. 3 shows the forest plot of meta-analysis for boys. The pooled rate of female cases was 40% (95% CI: 39%–41%). The male-to-female ratio was 1.5:1.

## Discussion

In China, recurring outbreaks of HFMD among preschool children is a major public health concern. To the best of our knowledge, this is the first meta-analysis to identify epidemiological features of HFMD outbreaks in preschools across China. Month, region, age, and gender might be used as risk indicators for HFMD outbreaks. This will provide guidance for HFMD prevention and help to reduce outbreaks of HFMD in pre-schools.

In temperate climate, HFMD outbreaks usually occur in warmer months of the year, whereas in tropical areas, HFMD epidemics increase in wet season (36). For example, in the USA, outbreaks of HFMD typically occur during summer and autumn months (37). In Taiwan, seasonal variations in HFMD incidence were observed, of which an incidence peak occurred in the summer months (38). In our study, outbreaks of HFMD peaked in late spring and summer months from Apr to Jul, similar to that found in other temperate regions.

Such peak month pattern might be partly explained by climatic factors, such as temperature and humidity.

In Japan, higher temperature could influence the increase of HFMD incidence (39). In addition, sentinel surveillance in Belgium has shown that HFMD viruses were more active in summer months when the temperature is high (40). A Singapore study pointed out that a maximum daily temperature above 32 °C and rainfall up to 75 mm was expected to increase the HFMD incidence (36). In China, the average temperature and humidity are relatively high in the period of Apr to Jul. The environment is warm and damp, which are conducive to the survival of enteroviruses and the transmission of HFMD (41–43). Considering the peak month pattern of HFMD outbreaks, we recommend that during the period of Apr–Jul each year, routine epidemiological surveillance should be strengthened among pre-school children.

The difference in HFMD incidence between urban and rural areas has also been discussed in other studies. The rural region in Taiwan had the least incidences of severe HFMD cases (38). Children living in urban areas and in rural areas accounted for 70.5% and 29.5% of the severe HFMD cases respectively (44). In our study, HFMD outbreaks occurred more in urban pre-schools than those in rural areas. High population density and the congregation of migrant children might be two underlying causes. Previous studies have proved a correlation between the density of children and the incidence of HFMD (45, 46). In urban areas of China, pre-school children commonly congregate in limited space, which provides an available reservoir for the rapid transmission of the virus. Additionally, urban areas in China accommodate lots of migrant children. Migrant children were at significantly high risk of developing HFMD (47). Migrant children had a higher HFMD attack rate than permanent residents (48). Terrible sanitation, tardy care, and poor educational environment may result in the susceptibility of migrant children. We suggest that public health measures of controlling HFMD outbreaks should better focus on urban areas preferentially.

Age has been identified as a risk factor for HFMD infection in previous studies. Data from Asia countries showed that HFMD incidence rate varies widely even within the narrow 0- to 5-yr age-band. A serologic survey in Singapore children showed that seropositivity to EV71 increases rapidly from age 2 to 5 yr (49). In Malaysia, HFMD incidence was the highest in children <2 yr and decreased with age (50). An 8-year study of epidemiologic features of EV71 infection in Taiwan found that younger children (< 4 yr of age) are at an apparently increased risk for HFMD infection (51). In China, younger children less than 5 yr accounted for the majority of sporadic cases (52–54). Our study confirmed the impact of age on HFMD infection but paid more attention to the differences between each age group among preschool children in HFMD outbreaks. In our study, children aged 37–48 months appeared to be the most susceptible age group during HFMD outbreaks. We recommend that special preventive measures should be taken in the future to protect this age group of children.

Another interesting finding in our study was that gender might be a risk factor of HFMD outbreaks among preschool children. The male-to-female ratio was 1.5:1. In a report from Singapore, there was a male predominance of HFMD cases, with a male-to-female ratio between 1.3:1 and 1.6:1 (55). In Taiwan, during the enterovirus outbreak in 2008, the male-to-female ratio was 1.6:1 (56). These findings might suggest susceptibility at the host genetic level. However, the reasons are unclear and deserve further research.

## Conclusion

Month, residence, age and gender might be early risk factors for potential HFMD outbreaks. To control future HFMD outbreaks among pre-school children, prevention measures should be taken before the advent of peak months from Apr to Jul each year. Those preschools located in urban areas should take priority over preventing and controlling the disease. Further professional protection should be given to high-risk children 37–48 months old, especially preschool boys. We hope that data from China could help other countries to improve the quality of HFMD prevention.

## Ethical considerations

Ethical issues (Including plagiarism, informed consent, misconduct, data fabrication and/or falsification, double publication and/or submission, redundancy, etc.) have been completely observed by the authors.

## References

[1] World Health Organization (2011). A Guide to Clinical Management and Public Health Response for Hand, Foot and Mouth Disease (HFMD). Geneva: WHO Press http://www.wpro.who.int/emerging_diseases/documents/HFMDGuidance/en/

[2] Pathinayake PS Hsu AC Wark PA (2015). Innate Immunity and Immune Evasion by Enterovirus 71. Viruses, 7(12): 6613–30. 26694447 10.3390/v7122961 PMC4690884

[3] Xie YH Chongsuvivatwong V Tan Y (2015). Important roles of public playgrounds in the transmission of hand, foot, and mouth disease. Epidemiol Infect, 143(7): 1432–41. 25170900 10.1017/S0950268814002301 PMC9507205

[4] Hosoya M Kawasaki Y Sato M (2006). Genetic diversity of enterovirus 71 associated with hand, foot and mouth disease epidemics in Japan from 1983 to 2003. Pediatr Infect Dis J, 25(8): 691–4. 16874167 10.1097/01.inf.0000227959.89339.c3

[5] Chan LG Parashar UD Lye MS (2000). Deaths of children during an outbreak of hand, foot, and mouth disease in Sarawak, Malaysia: clinical and pathological characteristics of the disease. Clin Infect Dis, 31(3): 678–83. 11017815 10.1086/314032

[6] Chan K Goh K Chong C (2003). Epidemic hand, foot and mouth disease caused by human enterovirus 71. Emerg Infect Dis, 9(1): 78–85. 12533285 10.3201/eid1301.020112 PMC2873753

[7] Geoghegan JL Tan le V Kühnert D (2015). Phylodynamics of Enterovirus A71-Associated Hand, Foot, and Mouth Disease in Viet Nam. J Virol, 89(17): 8871–9. 26085170 10.1128/JVI.00706-15 PMC4524079

[8] Chen Q Lin J Li Y Wei L Zhuang JL (2014). Analysis of the epidemiological characteristics of hand-foot-mouth disease epidemics in Beixinjing, Changning district of Shanghai during 2010 to 2012. Capital Journal of Public Health, 8(2): 65–67.

[9] Zhuang ZC Kou ZQ Bai YJ (2015). Epidemiological Research on Hand, Foot, and Mouth Disease in Mainland China. Viruses, 7(12): 6400–11. 26690202 10.3390/v7122947 PMC4690870

[10] Xing W Liao Q Viboud C (2014). Hand, foot, and mouth disease in China, 2008–12: an epidemiological study. Lancet Infect Dis, 14(4): 308–18. 24485991 10.1016/S1473-3099(13)70342-6 PMC4035015

[11] National Health and Family Planning Commission of China (2016). National incidence and death cases of notifiable communicable diseases. www.nhfpc.gov.cn/jkj/pgzdt/list.shtml

[12] Xu HM Zhu WP Wan Y (2014). Investigation on influencing factors of cluster onset of hang-foot-and-mouth disease in kindergartens of Pudong New Area in Shanghai city. Chin Matern Child Health J, 29: 4130–3.

[13] Ministry of Health of the People’s Republic of China (2009). Hand, foot, and mouth disease prevention and control guideline. www.gov.cn/gzdt/2009-06/04/content_1332078.htm

[14] Stroup DF Berlin JA Morton SC (2000). Meta-analysis of observational studies in epidemiology: a proposal for reporting. Meta-analysis of Observational Studies in Epidemiology (MOOSE) group. JAMA, 283(15): 2008–12. 10789670 10.1001/jama.283.15.2008

[15] Peng RR Wang AL Li J (2011). Molecular typing of Treponema pallidum: a systematic review and meta-analysis. PLoS Negl Trop Dis, 5(11): e1273. 22087340 10.1371/journal.pntd.0001273 PMC3210736

[16] Han TJ Li JS Wang L Xv H (2016). Coffee and the Risk of Lymphoma: A Meta-analysis Article. Iran J Public Health, 45(9): 1126–35. 27957457 PMC5149466

[17] Li M Zhao Y Duan K (2012). Epidemiological characteristics of clustering cases of hand, foot and mouth disease in child care settings in Licheng, Jinan. Dis Surveil, 2(7): 524–6.

[18] Kong ZF (2014). Epidemiological characteristics of clustering and clusters of hand-foot-mouth disease in child care institutions in Ninghai, Zhejiang during 2012. Modern Prev Med, 41(13): 2372–4.

[19] Wang RQ Li HT Cai X (2013). Retrospective analysis of HFMD clusters in preschools in Changping district. Pract Prev Med, 20(8): 963–4.

[20] Zhang ZW Yang QD Huang XH (2014). Epidemiological analysis on 7 clusterss of hand, foot and mouth disease in Kindergartens in Dongguan City. Pract Prev Med, 21(6): 678–80.

[21] Li Z Xu HR Cheng HQ (2013). Epidemiological analysis of hand-foot-mouth disease clustering among Jinan kindergartens in 2012. Chin J Sch Health, 34(7): 851–2.

[22] Tian L Zhu RY Fan JH (2011). Epidemiological analysis of hand-foot-mouth disease clustered cases in kindergartens in Shanghai during 2009. Chin J Sch Health, 32(5): 596–8.

[23] Wang LJ Gao H Wang J (2013). Epidemiological analysis and control measure of 27 gathering epidemics of HFMD. Modern Prev Med, 40(9): 1601–4.

[24] Ye ZW Ruan F Zhang XB (2014). Analysis of epidemiological features of hand, foot and mouth disease (HFMD) in nurseries of Zhuhai city. Chin Trop Med, 14(4): 414–6.

[25] Xv JJ He F Yang SH (2013). Epidemic characteristics of HFMD in nurseries of Yandu district of Yancheng city. Occup and Health, 29(23): 3126–8.

[26] Chen HQ Xu HR Chang CY (2010). Epidemiological investigation of 5 clusters of hand foot mouth disease (HFMD) at the nurseries or kindergartens of Jinan. Chin J Sch Health, 31(7): 828–9.

[27] Gu YG Duan SB (2010). Analysis on the epidemic of Hand-foot-mouth Disease among kindergartens of Shizhong district, Jining city. Prev Med Trib, 16(8): 735–7.

[28] Hu QP Wang XQ Yin W (2015). Influence on cases clustering of hand, foot and mouth disease of initial case in kindergartens in Pudong district of Shanghai. Chin Prev Med, 16(2): 108–11.

[29] Wang J Xu MG Li EG Zhou Z (2013). Analysis of the epidemiological characteristics of hand-foot-mouth disease clustered cases in Zhabei district of Shanghai. Modern Prev Med, 40(14): 2577–80.

[30] Zhang CD Ying L (2013). Epidemiological characteristics of hand foot and mouth disease clusters in Lianyungang during 2010–2011. Jiangsu J Prev Med, 24(1): 23–5.

[31] Zhao ZR Yao WL Chen J Cheng XF (2013). Analysis of the epidemiological characteristics of clustered cases of hand, foot and mouth disease in Maanshan from 2010 to 2011. J Patho Biolo, 8(2): 164–6.

[32] Xiong JG Liu YZ Zeng YM (2012). Analysis on the epidemic situation of hand foot and mouth disease in Dongguan. J Trop Med, 12(4): 486–9.

[33] Li JY Mai BG Cao XO (2012). Analysis of hand foot and mouth disease clusters and emergency response in Nanhai district of Foshan City. Chin Prev Me, 13(9): 706–7.

[34] Wang JP Du DF Xu XC (2013). Analysis of infectious disease clusters among schools and nurseries in Yidu city in 2010. J Prev Med Inf, 29(1): 71–2.

[35] Liu Y Qin L Teng YZ Meng XH (2015). Analysis on aggregation clusterss of hand-foot-mouth disease in kindergartens of Dandong city from 2009 to 2013. Occup and Health, 31(3): 390–2.

[36] Hii YL Rocklov J Ng N (2011). Short term effects of weather on hand, foot and mouth disease. PLoS One, 6(2): e16796. 21347303 10.1371/journal.pone.0016796 PMC3037951

[37] Centers for Disease Control and Prevention (CDC) (2012). Notes from the field: severe hand, foot, and mouth disease associated with coxsackievirus A6-Alabama, Connecticut, California, and Nevada, November 2011–February 2012. MMWR Morb Mortal Wkly Rep, 61(12): 213–4. 22456122

[38] Chen KT Chang HL Wang ST (2007). Epidemiologic features of hand-foot-mouth disease and herpangina caused by enterovirus 71 in Taiwan, 1998–2005. Pediatrics, 120(2): e244–52. 17671037 10.1542/peds.2006-3331

[39] Urashima M Shindo N Okabe N (2003). Seasonal models of herpangina and hand-foot-mouth disease to simulate annual fluctuations in urban warming in Tokyo. Jpn J Infect Dis, 56(2): 48–53. 12824684

[40] Druyts-Voets E (1997). Epidemiological features of entero non-poliovirus isolations in Belgium 1980–94. Epidemiol Infect, 119(1): 71–7. 9287946 10.1017/s0950268897007656 PMC2808825

[41] Yin MM Yang JX Yang Y (2015). Analysis on the epidemiological characteristics of hand-foot-mouth disease clustered cases in hongkou district of Shanghai. Chinese Primary Health Care, 29(5): 89–90.

[42] Qiaoyun F Xiongfei J Lihuan L Angao X (2013). Epidemiology and etiological characteristics of hand, foot and mouth disease in Huizhou City between 2008 and 2011. Arch Virol, 158(4): 895–9. 23229012 10.1007/s00705-012-1566-6

[43] Xu M Yu W Tong S (2015). Non-Linear Association between Exposure to Ambient Temperature and Children’s Hand-Foot-and-Mouth Disease in Beijing, China. PLoS One, 10(5): e0126171. 26010147 10.1371/journal.pone.0126171 PMC4444089

[44] Zeng M Pu D Mo X (2013). Children of rural-to-urban migrant workers in China are at a higher risk of contracting severe hand, foot and mouth disease and EV71 infection: a hospital-based study. Emerg Microbes Infect, 2(10): e72. 26038441 10.1038/emi.2013.72 PMC3826070

[45] Huang J Wang J Bo Y (2014). Identification of health risks of hand, foot and mouth disease in China using the geographical detector technique. Int J Environ Res Public Health, 11(3): 3407–23. 24662999 10.3390/ijerph110303407 PMC3987041

[46] Hu M Li Z Wang J (2012). Determinants of the incidence of hand, foot and mouth disease in China using geographically weighted regression models. PLoS One, 7(6): e38978. 22723913 10.1371/journal.pone.0038978 PMC3377651

[47] Ge Y Zheng Y Pan H (2015). Epidemiological surveillance of hand, foot and mouth disease in Shanghai, 2010–2014. Zhonghua Er Ke Za Zhi, 53(9): 676–83. 26757968

[48] Ruan F Yang T Ma H (2011). Risk factors for hand, foot, and mouth disease and herpangina and the preventive effect of hand-washing. Pediatrics, 127(4): e898–904. 21422083 10.1542/peds.2010-1497

[49] Ooi EE Phoon MC Ishak B (2002). Seroepidemiology of human enterovirus 71, Singapore. Emerg Infect Dis, 8(9): 995–7. 12194783 10.3201/eid0809.10.3201/eid0809.010397 PMC2732542

[50] NikNadia N Sam IC Rampal S (2016). Cyclical Patterns of Hand, Foot and Mouth Disease Caused by Enterovirus A71 in Malaysia. PLoS Negl Trop Dis, 10(3): e0004562. 27010319 10.1371/journal.pntd.0004562 PMC4806993

[51] Chen SC Chang HL Yan TR (2007). An eight-year study of epidemiologic features of enterovirus 71 infection in Taiwan. Am J Trop Med Hyg, 77(1): 188–91. 17620652

[52] Ma E Chan KC Cheng P (2010). The enterovirus 71 epidemic in 2008--public health implications for Hong Kong. Int J Infect Dis, 14(9): e775–80. 20599410 10.1016/j.ijid.2010.02.2265

[53] Huang X Wei H Wu S (2015). Epidemiological and etiological characteristics of hand, foot, and mouth disease in Henan, China, 2008–2013. Sci Rep, 5:8904. 25754970 10.1038/srep08904 PMC4354091

[54] Wang X Wu X Jia L (2014). Estimating the number of hand, foot and mouth disease amongst children aged under-five in Beijing during 2012, based on a telephone survey of healthcare seeking behavior. BMC Infect Dis, 14:437. 25117760 10.1186/1471-2334-14-437 PMC4149051

[55] Ang LW Koh BK Chan KP (2009). Epidemiology and control of hand, foot and mouth disease in Singapore, 2001–2007. Ann Acad Med Singapore, 38(2): 106–12. 19271036

[56] Chen SP Huang YC Li WC (2010). Comparison of clinical features between coxsackievirus A2 and enterovirus 71 during the enterovirus outbreak in Taiwan, 2008: a children’s hospital experience. J Microbiol Immunol Infect, 43(2): 99–104. 20457425 10.1016/S1684-1182(10)60016-3

